# Supplementary material for: Inflammation and Treatment-Resistant Depression from Clinical to Animal Study: A Possible Link?
Source: Neurol Int. 2023 Jan 16;15(1):100–20. doi: 10.3390/neurolint15010009 (PMC9844360; doi:10.3390/neurolint15010009)
Supplement: Supplementary file 1 [file neurolint-15-00009-s001.zip › neurolint-2045967-supplementary.pdf]

brain sciences

MDPI

Supplementary

Table S1. Descriptive Statistics for the Study Sample.

		Frequency	Percent
Group	Respondent	39	49.3
	Resistant	40	50.7
Gender	Female	55	69.4
	Male	24	30.6
Education	No	35	56.6
	Yes	44	43.4
Smoking	No	71	91.7
	Yes	8	8.3
Inflammatory disease	No	19	12.9
	Yes	60	87.1
Chronic Disease	No	19	12.9
	Yes	60	87.1
Currently Taking AD	No	7	8.1
	Yes	72	91.9
Improvement with medications	No	39	56.5
	Yes	40	43.5
Hospitalization	No	73	83.9
	Yes	6	16.1
Family History	No	67	87.1
	Yes	12	12.9
Suicide	No	74	87.1
	Yes	5	12.9
Repeated visits	No	65	40.3
	Yes	14	59.7
Change of AD in cthe urrent year	No	47	62.9
	Yes	32	37.1
Adherence	No	22	11.3
	Yes	57	88.7

AD: antidepressant.

Table S2. Comparison of the Treatment Respondent Group vs. Treatment Resistant Group in the Demographic and Clinical Data.

		Group		P-value
		Respondent	Resistant	
Gender	Female	24<br>54.5%	31<br>77.5%	0.061
	Male	15<br>45.5%	9<br>22.5%	
Education	No	11<br>40.0%	24<br>63.2%	0.125
	Yes	28<br>60.0%	14<br>36.8%	
Smoking	No	36<br>90.9%	35<br>92.1%	>0.999
	Yes	3<br>9.1%	3<br>7.9%	
Inflammatory disease	No	15<br>18.2%	4<br>10.0%	0.438
	Yes	24<br>81.8%	36<br>90.0%	
Chronic Disease	No	14<br>13.6%	5<br>12.5%	>0.999
	Yes	25<br>86.4%	35<br>87.5%	
Taking AD	No	6<br>18.2%	1<br>2.5%	0.049
	Yes	33<br>81.8%	39<br>97.5%	
Improvement with medications	No	0<br>0.0%	35<br>87.5%	<0.001
	Yes	39<br>100.0%	5<br>12.5%	
Hospitalization	No	38<br>95.5%	31<br>77.5%	0.082
	Yes	1<br>4.5%	9<br>22.5%	
Family History	No	36<br>86.4%	35<br>87.5%	>0.999
	Yes	3<br>13.6%	5<br>12.5%	
Suicide	No	39<br>100.0%	32<br>80.0%	0.042

	Yes	0	8	
		0.0%	20.0%	
Repeated visits	No	33	9	<0.001
		72.7%	22.5%	
	Yes	6	31	
		27.3%	77.5%	
Change of AD in current year	No	38	18	<0.001
		95.5%	45.0%	
	Yes	1	22	
		4.5%	55.0%	
Adherence	No	35	3	0.002
		18.2%	7.5%	
	Yes	4	37	
		81.8%	92.5%	
Age Mean (SD)		51.5(17.3)	49.8(15.3)	0.481
BMI Mean (SD)		33.7(9.2)	31.3(7.6)	0.205

AD: antidepressants; BMI: body mass index; SD: standard deviation.

The variables that differentiated the treatment-resistant patients from the treatment responders were statistically significant between both groups. AD use (P<0.049), improvement with medications (P<0.001), suicide (P<0.042), repeated visits (P<0.001), changes in AD in the current year (P<0.001), and adherence (P<0.002).

Table S3. Comparison of the Laboratory Test Results between the Healthy Group vs. Depressed Group.

Group	Healthy			Depression			P-value
	N	Mean	SD	N	Mean	SD	
CRP	80	6.5	11.5	67	57.1	82.7	<0.001
ESR	75	30.6	22.7	64	44.7	31.3	0.004
WBC	102	7.5	2.4	99	8.9	4.8	0.007

CRP, C-reactive protein; ESR, erythrocyte sedimentation rate; WBC, white blood cell count. The levels of inflammatory biomarkers (CRP, ESR, and WBC) were significantly different between the groups: CRP (P <0.001), ESR (P = 0.004), and WBC (P=0.007).

Table S4. Comparison of the Laboratory Test Results between the Respondent Group vs. Resistant Group.

**Group**	**Respondent**						**Resistant**						**P-value**
	N	Mean	SD	Q1	Median	Q3	N	Mean	SD	Q1	Median	Q3	
**CRP**	23	11.08	7.7	4.2	1.8	15.6	28	61.0	97.0	5.2	15.4	83.6	0.031
**ESR**	21	27.2	12.4	8.0	5.0	53.0	22	50.7	31.4	30.0	43.0	64.3	0.048
**WBC**	28	8.7	4.6	4.8	5.3	7.9	38	7.9	3.2	5.2	7.2	11.1	0.174

CRP, C-reactive protein; ESR, erythrocyte sedimentation rate; WBC, white blood cell count. The inflammatory biomarkers (CRP and ESR) showed a statistically significant difference between the groups: CRP (P = 0.031) and ESR (P = 0.048).

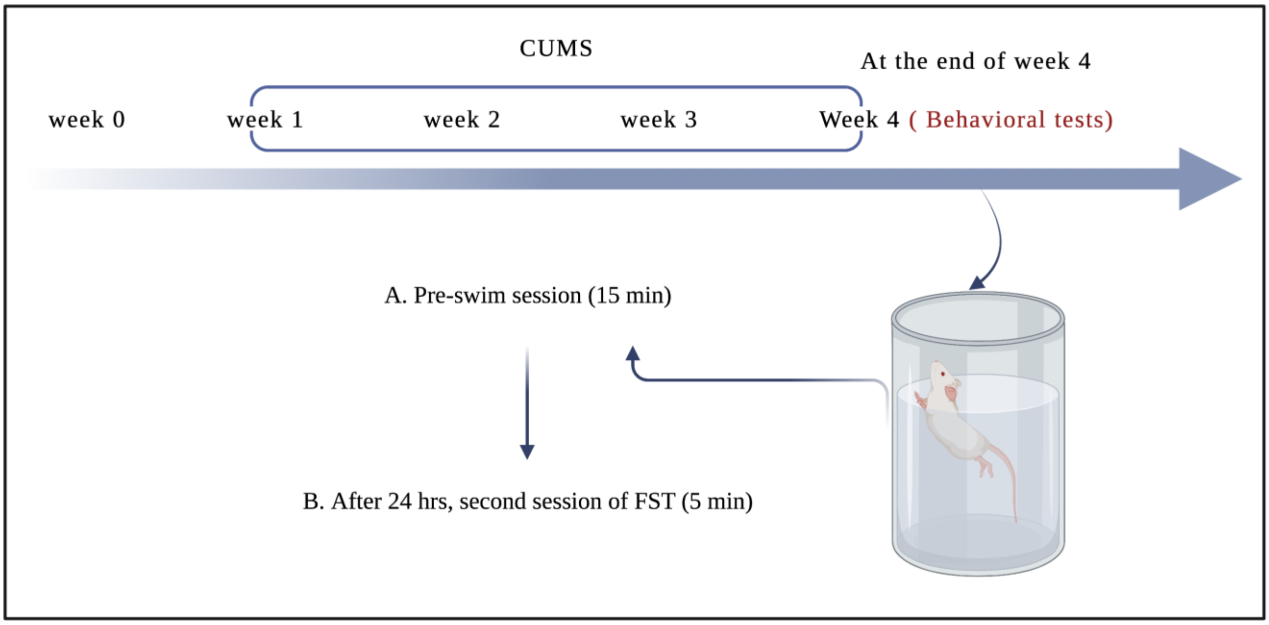
CUMS
At the end of week 4
week 0
week 1
week 2
week 3
Week 4 (Behavioral tests)
A. Pre-swim session (15 min)
B. After 24 hrs, second session of FST (5 min)

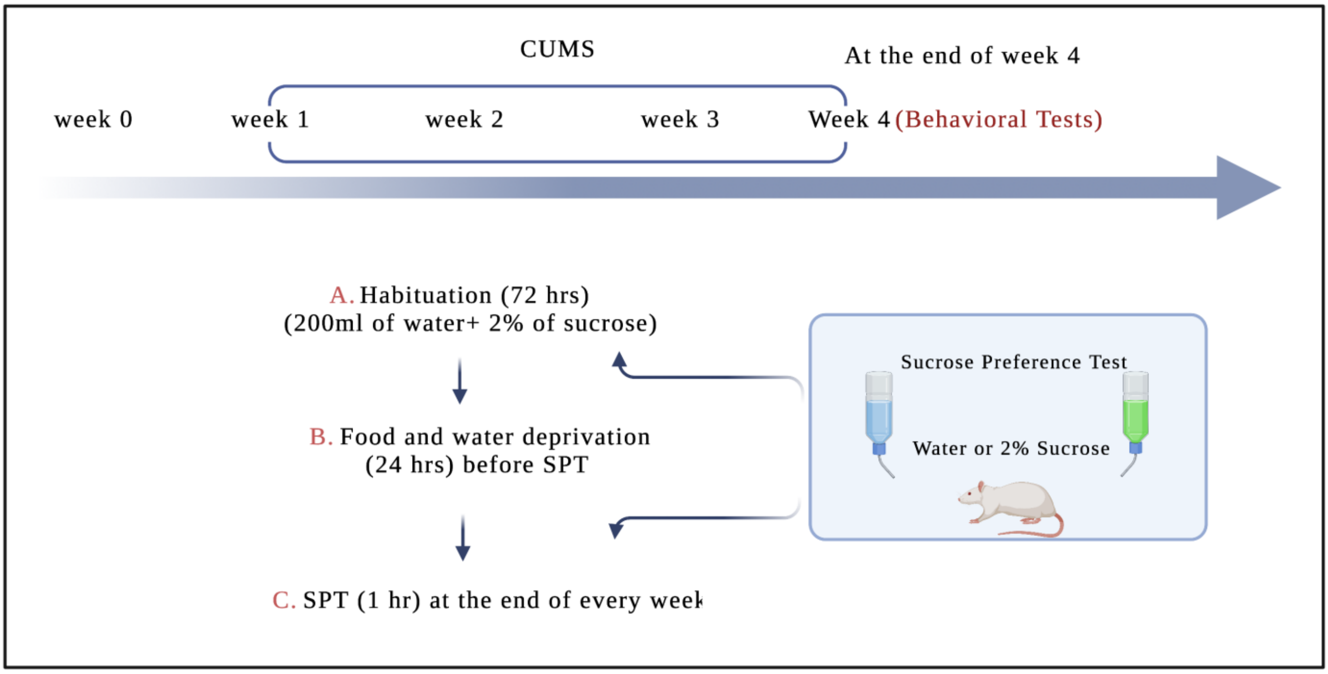
CUMS
At the end of week 4
week 0
week 1
week 2
week 3
Week 4 (Behavioral Tests)
A. Habituation (72 hrs)
(200ml of water+ 2% of sucrose)
B. Food and water deprivation
(24 hrs) before SPT
C. SPT (1 hr) at the end of every week
Sucrose Preference Test
Water or 2% Sucrose

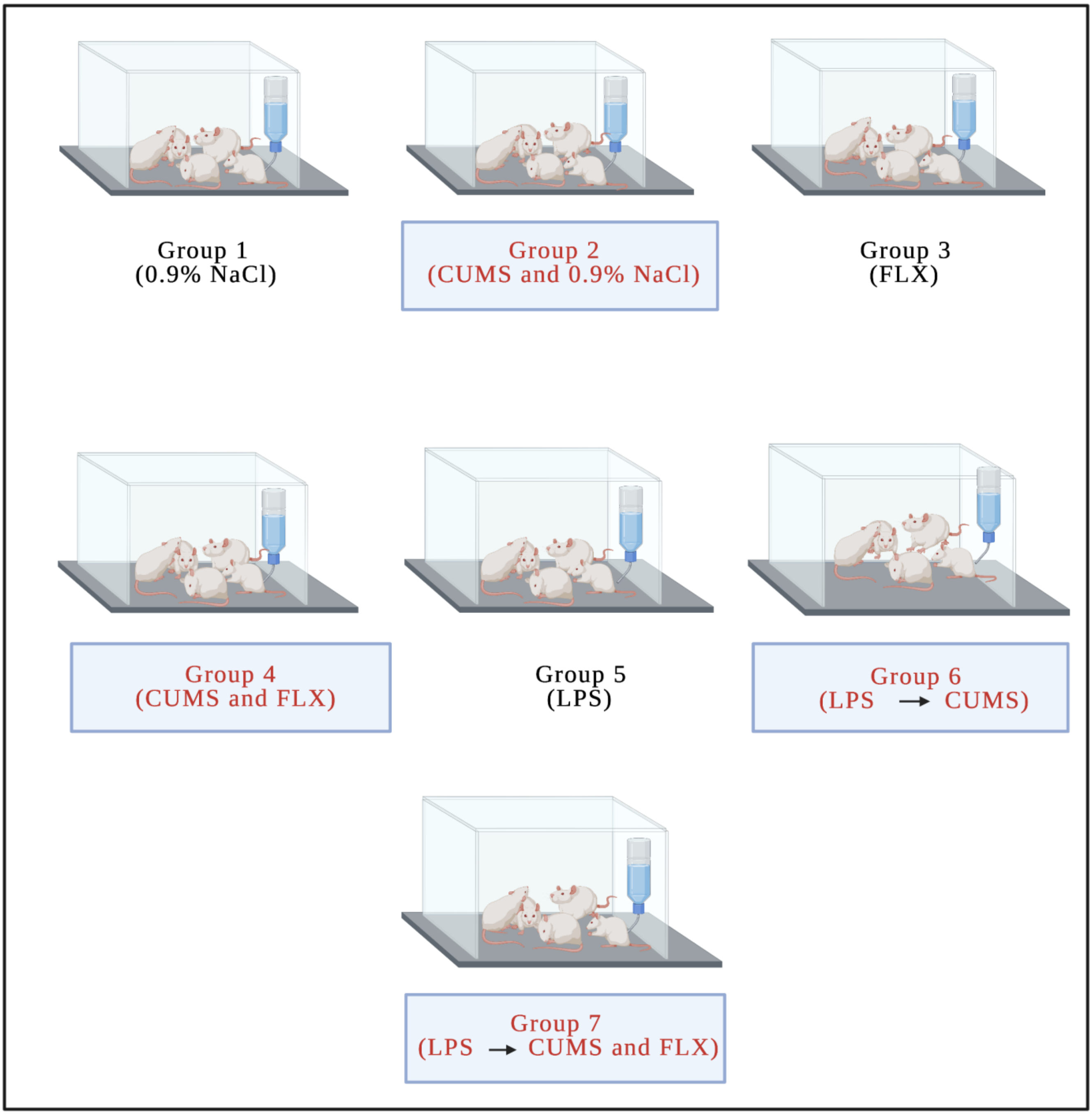
Group 1
(0.9% NaCl)
Group 2
(CUMS and 0.9% NaCl)
Group 3
(FLX)
Group 4
(CUMS and FLX)
Group 5
(LPS)
Group 6
(LPS → CUMS)
Group 7
(LPS → CUMS and FLX)

Figure S1.
